# Subconcussive changes in youth football players: objective evidence using brain vital signs and instrumented accelerometers

**DOI:** 10.1093/braincomms/fcab286

**Published:** 2021-12-15

**Authors:** Shaun D Fickling, Daniel N Poel, Jason C Dorman, Ryan C N D’Arcy, Thayne A Munce

**Affiliations:** 1 Faculty of Sciences and Applied Sciences, Simon Fraser University, Burnaby, BC V5A 1S6, Canada; 2 BrainNET, Health and Technology District, Surrey, BC V3V 0C6, Canada; 3 Center for Neurology Studies, HealthTech Connex, Surrey, BC V3V 0C6, Canada; 4 Sanford Sports Science Institute, Sanford Health, Sioux Falls, SD 57107, USA; 5 Environmental Influences on Health and Disease Group, Sanford Research, Sioux Falls, SD 57104, USA

**Keywords:** concussion, subconcussion, football, EEG, ERP

## Abstract

Brain vital signs, measured by EEG, were used for portable, objective, neurophysiological evaluation of cognitive function in youth tackle football players. Specifically, we investigated whether previously reported pre- and post-season subconcussive changes detected in youth ice hockey players were comparably detected in football. The two objectives were to: (i) replicate previously published results showing subconcussive cognitive deficits; and (ii) the relationship between brain vital sign changes and head-impact exposure. Using a longitudinal design, 15 male football players (age 12.89 ± 0.35 years) were tested pre- and post-season, with none having a concussion diagnosis during the season. Peak latencies and amplitudes were quantified for Auditory sensation (N100), Basic attention (P300) and Cognitive processing (N400). Regression analyses tested the relationships between these brain vital signs and exposure to head impacts through both number of impacts sustained, and total sessions (practices and games) participated. The results demonstrated significant pre/post differences in N400 latencies, with ∼70 ms delay (*P* < 0.01), replicating prior findings. Regression analysis also showed significant linear relationships between brain vital signs changes and head impact exposure based on accelerometer data and games/practices played (highest *R* = 0.863, *P* < 0.001 for overall sessions). Number of head impacts in youth football (age 12–14 years) findings corresponded most closely with prior Junior-A ice hockey (age 16–21 years) findings, suggesting comparable contact levels at younger ages in football. The predictive relationship of brain vital signs provided a notable complement to instrumented accelerometers, with a direct physiological measure of potential individual exposure to subconcussive impacts.

## Teaser

Brain vital signs, measured by EEG, were used for portable neurophysiological evaluation of cognitive function in a group of youth tackle football players. Fickling et al. observed strong, significant linear relationships between changes in brain vital signs and head-impact exposure (number of impacts, number of sessions) over the season.

## Graphical Abstract

**Graphical Abstract:**
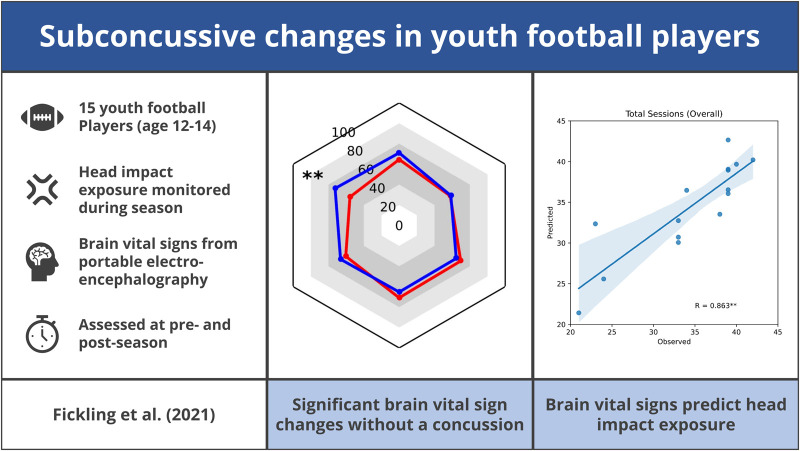

## Introduction

Tackle football is one of the most popular sports in the USA, with nearly 2.5 million participants, and most of the players (∼95%) are children and adolescents.^1–3^ Young players have both an extended window of injury exposure and their sport-related concussions tend to be more severe, with a longer period of recovery compared to adults.^4^ Emerging research has raised important questions about the safety of contact sport, particularly for children and adolescents.^5^

### Subconcussion in football

Sport-related concussions have been the focus of considerable research over the past two decades. More recently, there is growing interest in studying the effects of repetitive head impacts (RHI).^6,7^ Football players of all ages are subject to RHI, and growing evidence suggests that RHI-related trauma has relevance for both short- and long-term brain health. Acutely, cumulative head impact exposure (HIE) has been associated with changes in white matter activity and neurophysiological function in asymptomatic contact-/collision-sport athletes.^8,9^ Such observations have helped characterize the poorly understood phenomenon of subconcussion. Regarding long-term brain health, repetitive trauma (i.e. subconcussion) is the leading risk factor for the neurodegenerative disease chronic traumatic encephalopathy (CTE).^10^ Thus, early recognition of subconcussive RHI may help improve knowledge of the pathophysiology of trauma-related brain disease. However, subconcussive brain injury is currently poorly defined, and the identification of diagnostic markers remains elusive.

A central challenge in the evaluation of subconcussive impairment is the need for sensitive measures capable of detecting neurophysiological changes in brain function. Existing research in this area has largely relied upon anatomical and/or functional neuroimaging approaches [e.g. MRI, functional MRI, diffusion tensor imaging] to evaluate changes in brain structure and/or function. Advancements in point-of-care (PoC) testing are needed to enable more accessible monitoring of brain function to (i) better understand the mechanisms of subconcussive injury; (ii) limit the exposure to RHI; and (iii) inform treatment.

### Objective neurophysiological evaluation at PoC

The brain vital signs scientific framework was developed to address the need for an objective neurophysiological evaluation at PoC.^11^ In this framework, well-established cognitive evoked potentials, or event-related potentials (ERPs), are extracted from EEG signals through a brief (∼5 min) auditory stimulus sequence consisting of alternating tones and word pairs. The tone series consists of a pattern of standard tones in which unexpected deviant tones (louder tones) are infrequently embedded (i.e. oddball^12^ ). Deviant tones elicit well-established ERP responses called the N100 and P300, corresponding to auditory sensory processing and basic attention processing, respectively.^13,14^ To evoke higher level cognitive processing responses, spoken word pair stimuli are interleaved into the tone stimulus train at regular intervals. The word pairs are semantically primed through related (e.g. *pizza-cheese*) and unrelated (*pizza-window*) word pairs to elicit the N400 response.^15^ For a full description of the brain vital signs framework including the research behind the individual components, refer to Ghosh Hajra et al.^11^

### Brain vital sign and subconcussive impacts

Prior research in Junior-A ice hockey athletes has highlighted the sensitivity of brain vital signs to detect abnormalities following a diagnosed concussion.^16^ All six brain vital sign metrics demonstrated significant deviation from baseline after concussion. Notably, while the baseline pattern was largely re-established at return-to-play, significant residual impairments in basic attention were still detectable. These findings highlighted the sensitivity of brain vital signs to residual cognitive deficits that were undetected by clinical protocols. In a secondary analysis, it was observed that athletes who did not sustain a diagnosed concussion showed significant delays in the N400 measure of cognitive processing. This was interpreted to reflect neurophysiological abnormalities associated with RHI.^16^

To further examine these subconcussive changes in context of RHI, a follow-up study was conducted on male ice hockey players from both Bantam and Junior-A ages.^17^ Again, significant subconcussive changes were detected both across and within groups. Importantly, the subconcussive brain vital sign changes were significantly linearly related (*R* = 0.799, *P* > 0.01) to the number of head impacts experienced by players during the season. In addition, qualitative ERP waveform differences related to preceding perceptual and phonological speech processing were also reported. A sub-component preceding the N400, called the phonological mapping negativity (PMN^18,19^ ), appeared to partly account for the latency delays in the N400. It was noted that alterations in PMN appeared to be a common pattern across both studies.^17^

### Objectives and hypotheses

The current study focused on the relationship between subconcussive RHI and cognitive brain function in youth football players. Based on prior studies, we hypothesized that: (i) pre/post-season comparisons would reveal significant changes in brain vital signs; and (ii) brain vital signs would be linearly predictive of measures of RHI (number of impacts and sessions participated).

## Materials and methods

### Participants

Institutional review/ethics boards at Sanford Health and Simon Fraser University approved this study. Each participant provided written assent with parent/guardian consent, according to the declaration of Helsinki. Forty youth football players (39 male, 1 female; 13 ± 0.5 years) were studied over three separate seasons while participating in a community-based tackle football league. Each season lasted ∼ 3 months and consisted of 32 ± 1 practices and 9 ± 0 games. Six players were lost to follow-up, eight players withdrew from the study and three experienced a concussion during the season. There were eight additional players who completed pre- and post-season testing, but their data were not included due to poor signal quality and/or technical issues (broken electrode wire). Thus, final analysis included the remaining 15 players (12.89 ± 0.35 years) who completed pre- and post-season testing with sufficient data quality and were not concussed during their respective season. The average number of prior concussions in this group was 0.2 ± 0.4. Three (20%) players reported a single concussion occurring before their involvement in the study; none were symptomatic at the time of participation.

Sanford Health athletic trainers provided sports medicine services for the participants during games and on a walk-in basis throughout the season, including concussion diagnosis and management in accordance with current clinical practice guidelines. Data collected in this study were not used to inform any clinical treatment.

### Data collection

Pre-season assessments were completed the week before the start of each season. Post-season assessments were performed 1 week following the final game. EEG data were collected using a wireless eight-channel g. Nautilus system (Gtec Medical Engineering, Austria). Participants were asked to sit still and listen attentively to the audio sequence, but no active participation was required. To reduce blink activity, which contaminates the EEG signal, participants were instructed to look at a visual fixation cross positioned on a wall at eye-level ∼ 2 m away. Distractions were mitigated by performing the scans in a quiet, closed room. The same facility was used for pre-season and post-season testing. One 5-min EEG recording was collected per participant per time point.

Subjects’ HIE during all practices and games was measured using the Head Impact Telemetry (HIT) System (Riddell Corp, Elyria, OH). The HIT System consists of an encoder unit with six spring-mounted single-axis accelerometers, an onboard data acquisition (8 bit; 1000 Hz/channel) and memory storage device (up to 100 impacts) and a wireless transceiver (903–927 MHz). The HIT encoders were placed within Riddell Revolution Speed helmets. When one accelerometer registered an impact equal to or greater than a predefined threshold of 10 g, 40 ms (8 ms pre-trigger; 32 ms post-trigger) of data were transmitted from all six accelerometers to a laptop computer on the sideline. Estimations of resultant linear acceleration, rotational acceleration and impact location were generated by the HIT System using the programme’s built-in algorithms. Impacts that occurred during non-football activities (e.g. water breaks) were removed from the analysis. A member of the research team was present at all practices and games to monitor player activities.

### Data analysis

#### Preprocessing

A blinded reviewer generated brain vital signs from the raw EEG using a semi-automated method described in prior work.^17^ Data were excluded due to poor quality if the stimulus timing information was lost or >30% of available ERP epochs were rejected due to noise (amplitudes > ±75 uV). For data of sufficient quality, ERP peaks were identified as the maximal peaks occurring within expected temporal ranges. Amplitude and latency metrics from these peaks were then linearly transformed into standardized scores on a scale from 0 to 100, derived from entire group means ± 3 SDs.^16^ This transformation provides easily interpretable metrics for evaluating multivariate change-over-time and allows changes in all six brain vital signs to be presented on the same scale.

Head impact frequency, linear acceleration and rotational acceleration data for each player were acquired from the HIT System. Total games played and practices were also extracted from the accelerometer data as an alternate measure of HIE.

#### Statistical analysis

A repeated-measures analysis of variance (RM-ANOVA) was used to evaluate changes from pre-season to post-season across all brain vital sign metrics. Secondly, stepwise regression models were used to assess the relationship between changes in brain vital signs scores (i.e. post-pre) and the total number of impacts, and sessions participated in during the season. Separate models (i.e. six total) were generated for games, practices, and games and practices combined. Finally, a non-parametric bootstrapping method was used to evaluate changes at the ERP waveform level.^20^ Statistical analyses were completed using Python and R-studio.

### Data availability

Raw data were generated by investigators at Sanford Research. Derived data supporting the findings of this study can be made available from the corresponding author on request.

## Results

**Figure 1:**
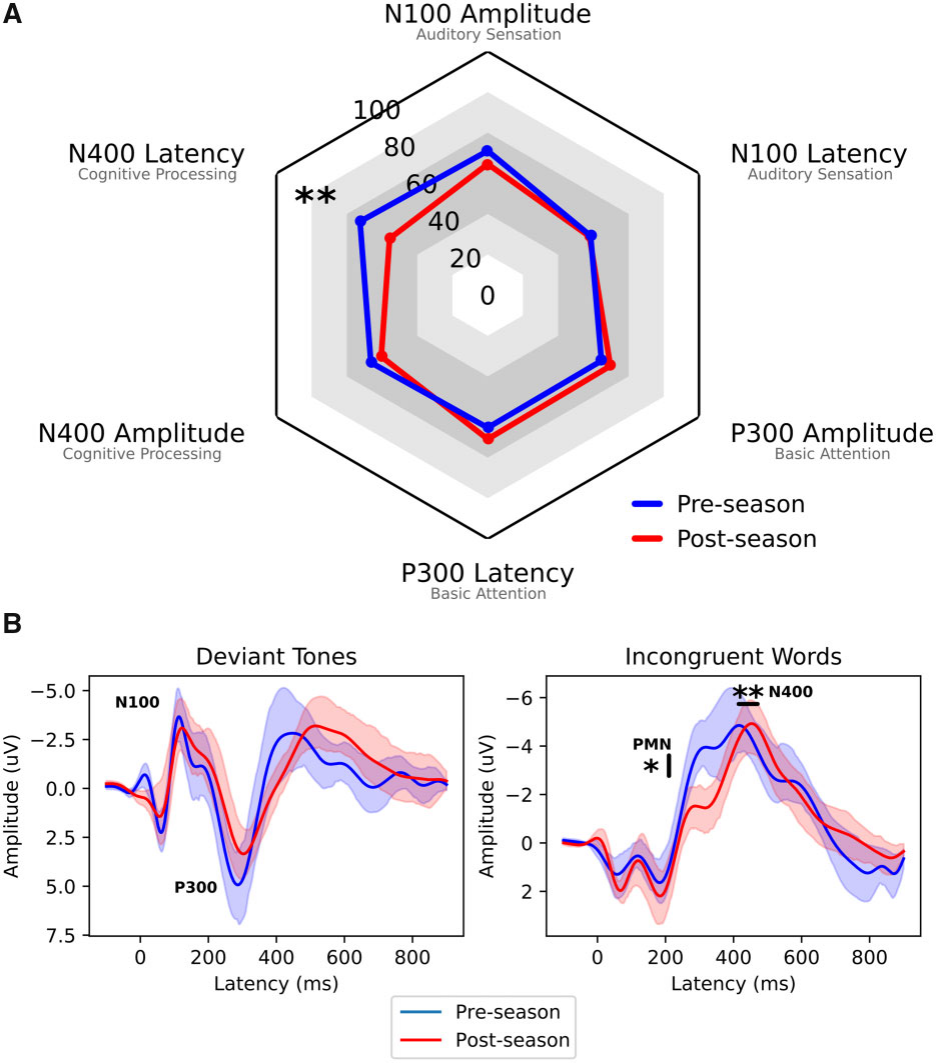
**Group mean results**. (**A**) Group radar plots showing significant delay in N400 latency on a standardized scale of 0–100 (**P < 0.01, test: RM-ANOVA). (**B**) Grand-average ERP waveforms (± 95% CI of the mean) for the deviant tone (left) and incongruent word (right) responses for pre- and post-season. N100, P300, PMN and N400 peaks are labelled on the Figure, showing the change in amplitude (*y*-axis) and latency (*x*-axis) over the course of the season. Significant differences between waveforms can be recognized by the separation between the 95% confidence intervals (*P < 0.05 test: non-parametric bootstrap).

Descriptive statistics for all brain vital signs and head impact variables are shown in Tables 1 and 2, respectively. In Table 1, brain vital signs are reported for both raw ERP values (amplitudes and latencies) and standardized brain vital signs scores. In Table 2, HIE metrics for total sessions, total number of impacts, median rotational acceleration and median linear acceleration are provided in means and standard deviations across the study group by games, practices and all sessions. The results of the RM-ANOVA are shown in Table 3. There were no significant multivariate changes, but a significant univariate change in N400 latency (*F* = 9.588, *P* = 0.01) was observed. Radar plots (Fig. 1A) of group mean scores are demonstrated alongside group mean ERP waveforms (Fig. 1B). In the radar plot format, longer (i.e. delayed) peak latencies and smaller peak amplitudes both result in lower standardized scores, highlighting the univariate change in the N400 latency component. ERP waveforms are shown for group mean average (±95% CI) of the deviant tone and incongruent word responses for pre- and post-season. N100, P300, PMN and N400 ERP peaks are labelled on the respective waveforms. The bootstrap permutation analysis revealed a significant effect for N400 amplitude around 300 ms post-stimulus at the group level (as shown by the separation in 95% confidence intervals in Fig. 1B).

**Table 1:** Descriptive statistics—brain vital signs

	Pre-season		Post-season	
	Raw values	Standardized scores	Raw values	Standardized scores
N1A	9.38 ± 3.85 uV	55.47 ± 18.13	8.31 ± 3.70 uV	50.43 ± 17.41
N1L	126.93 ± 23.47 ms	45.63 ± 18.22	127.60 ± 21.10 ms	45.11 ± 16.38
P3A	9.73 ± 3.82 uV	50.44 ± 15.04	10.74 ± 3.82 uV	54.39 ± 15.05
P3L	317.73 ± 86.61 ms	50.95 ± 18.06	296.67 ± 39.31 ms	55.34 ± 8.20
N4A	8.58 ± 2.92 uV	51.80 ± 16.43	7.77 ± 3.14 uV	47.20 ± 17.71
N4L	371.73 ± 53.69 ms	56.61 ± 12.58	427.73 ± 46.35 ms	43.50 ± 10.85

**Table 2:** Descriptive statistics—head impact exposure

	All games	All practices	Total (games + practices)
Sessions participated	8.00 ± 1.56	26.40 ± 5.41	34.40 ± 6.72
Total impacts	53.13 ± 39.91	80.00 ± 56.65	133.13 ± 85.64
Median rotational acceleration	1499.60 ± 201.41 rad/s^2^	1385.80 ± 217.95 rad/s^2^	1369.80 ± 151.11 rad/s^2^
Median linear Acceleration	20.81 ± 2.06 m/s^2^	19.53 ± 1.90 m/s^2^	19.83 ± 1.53 m/s^2^

**Table 3:** Repeated-measures MANOVA

	*F*-value	*P*
Multivariate comparisons		
Overall	2.391	0.116
Univariate comparisons		
N1A	1.478	0.244
N1L	0.010	0.922
P3A	1.161	0.299
P3L	2.129	0.167
N4A	0.799	0.387
N4L	9.207	0.009**

** *P* < 0.01,

**Table 4:** Summaries of the stepwise regression model results

Total sessions (overall)	***R***	***R* ^2^**	**Adj *R*^2^**	***F***	***P***
	0.863	0.744	0.674	10.67	0.001**
	**Coefficients**	**Estimate**	**Std error**	**T value**	***P***
	Intercept	31.211	1.360	22.946	<0.001**
	N100 Amplitude	0.297	0.073	4.048	0.002**
	N400 Amplitude	–0.203	0.061	–3.301	0.007**
	N400 Latency	–0.286	0.064	–4.469	0.001**
Total sessions (games only)	***R***	***R* ^2^**	**Adj *R*^2^**	***F***	***P***
	0.853	0.728	0.6193	6.693	0.007**
	**Coefficients**	**Estimate**	**Std error**	**T value**	***P***
	Intercept	7.211	0.371	19.456	<0.001**
	N100 Amplitude	0.063	0.020	3.331	0.008**
	P300 Latency	0.034	0.023	1.477	0.171
	N400 Amplitude	–0.036	0.015	–2.313	0.043*
	N400 Latency	–0.061	0.016	–3.753	0.004**
Total sessions (practices only)	***R***	***R* ^2^**	**Adj *R*^2^**	***F***	***P***
	0.841	0.707	0.6274	8.858	0.003**
	**Coefficients**	**Estimate**	**Std error**	***T*-value**	***P***
	Intercept	23.786	1.172	20.297	<0.001**
	N100 Amplitude	0.227	0.063	3.585	0.004**
	N400 Amplitude	–0.168	0.053	–3.174	0.009**
	N400 Latency	–0.228	0.055	–4.123	0.002**
Total impacts (overall)	***R***	***R* ^2^**	**Adj *R*^2^**	***F***	***P***
	0.747	0.559	0.382	3.165	0.064
	**Coefficients**	**Estimate**	**Std error**	***T*-value**	***P***
	Intercept	107.560	23.899	4.501	0.001**
	N100 Amplitude	3.514	1.301	2.700	0.022*
	N100 Latency	1.282	0.907	1.412	0.188
	N400 Amplitude	–1.770	1.084	–1.633	0.133
	N400 Latency	–2.732	1.127	–2.425	0.036*
Total impacts (games only)	***R***	***R* ^2^**	**Adj *R*^2^**	***F***	***P***
	0.823	0.677	0.498	3.78	0.040*
	**Coefficients**	**Estimate**	**Std error**	***T*-value**	***P***
	Intercept	44.713	11.298	3.958	0.003**
	N100 Amplitude	1.450	0.587	2.471	0.036*
	N100 Latency	1.345	0.455	2.956	0.016*
	P300 Latency	–1.043	0.814	–1.282	0.232
	N400 Amplitude	–0.777	0.455	–1.705	0.122
	N400 Latency	–1.330	0.474	–2.805	0.021*
Total impacts (practices only**)**	***R***	***R* ^2^**	**Adj *R*^2^**	***F***	***P***
	0.577	0.3324	0.221	2.988	0.089
	**Coefficients**	**Estimate**	**Std error**	***T*-value**	***P***
	Intercept	74.919	17.152	4.368	<0.001**
	N100 Amplitude	1.7106	0.831	2.058	0.062
	N400 Latency	–1.046	0.798	–1.31	0.214

* *P* < 0.05.

** *P* < 0.01.

**Figure 2:**
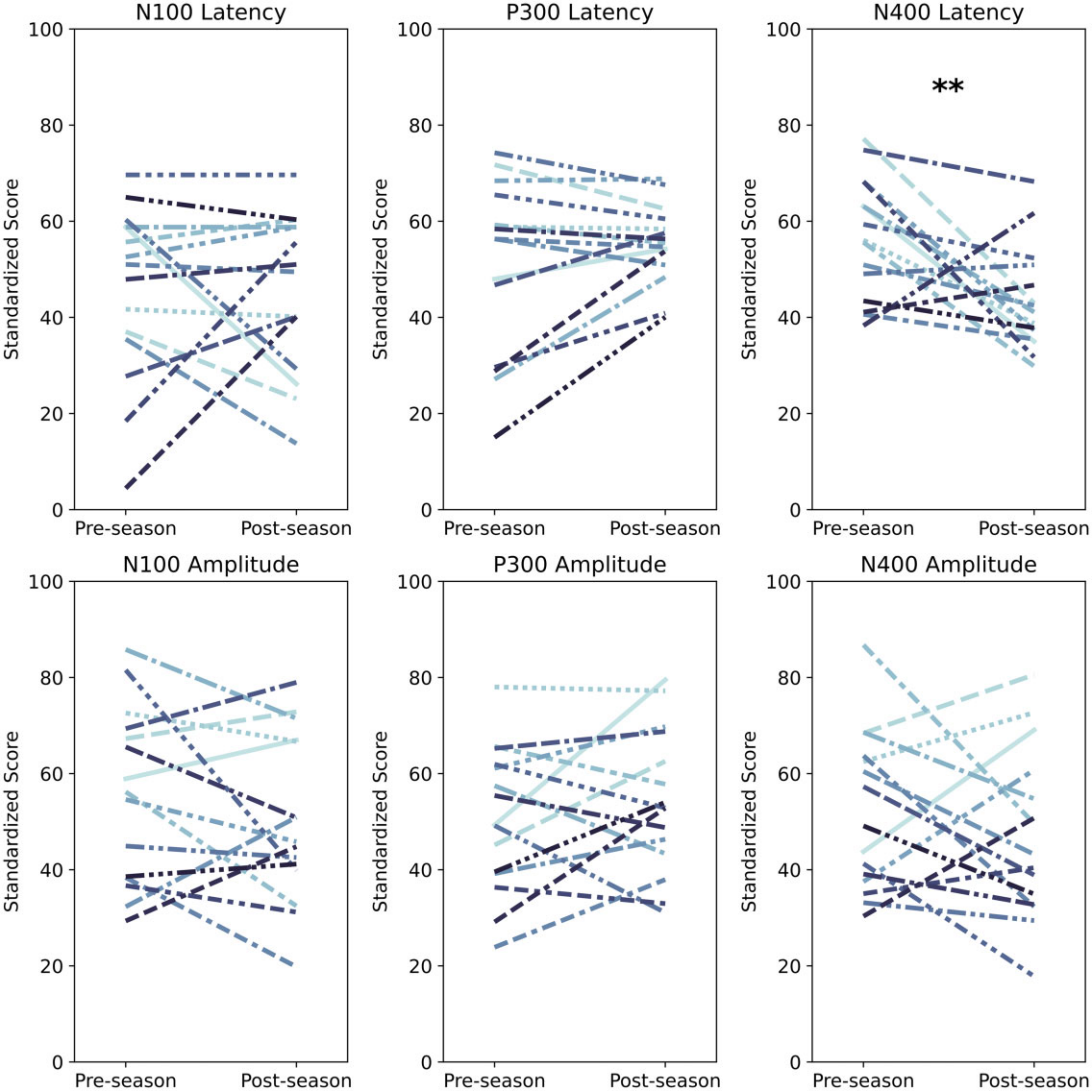
Individual changes (pre/post-differences) in brain vital sign scores for all 15 players. **P < 0.01, statistical test: RM-ANOVA.

**Figure 3:**
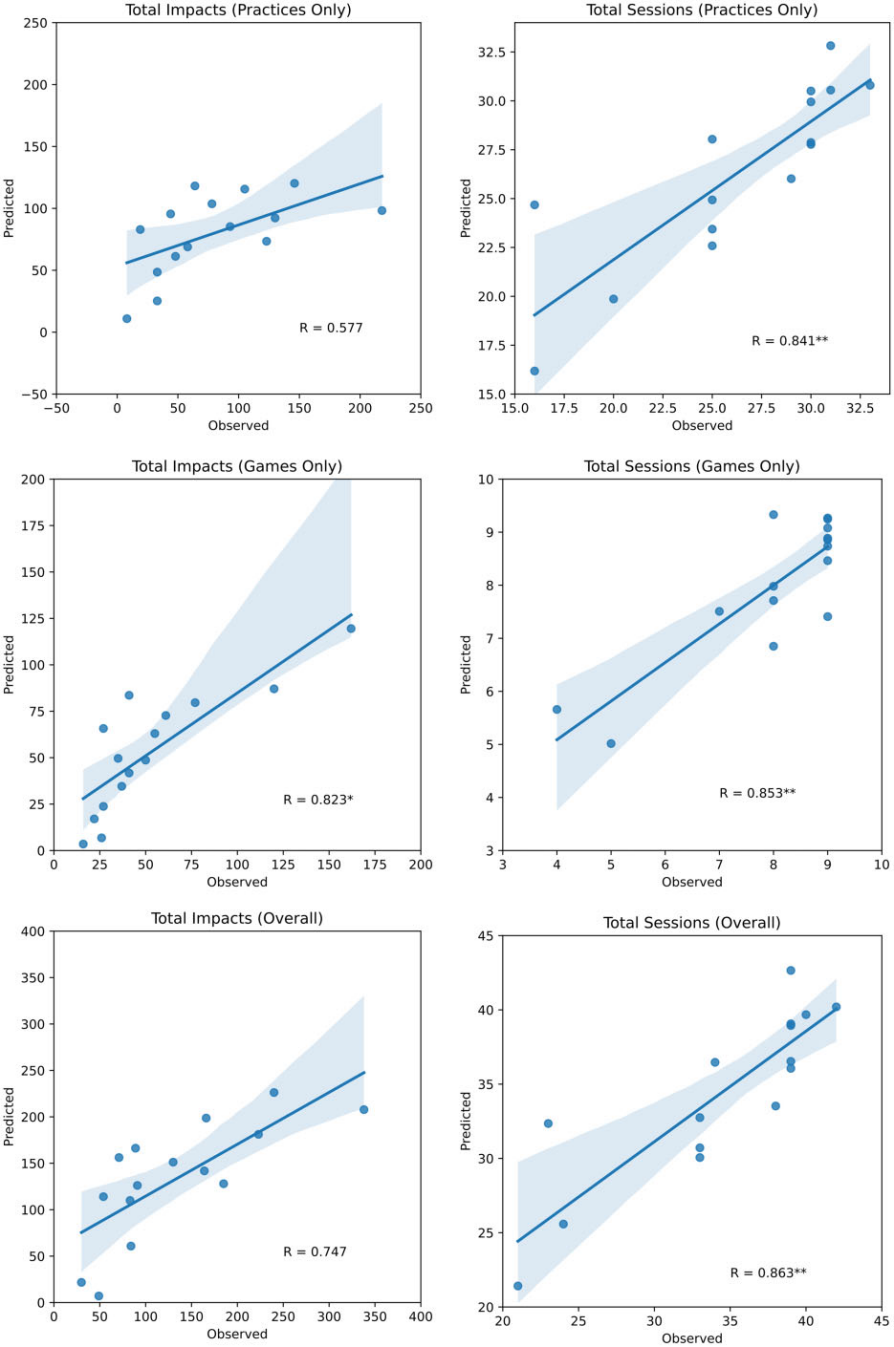
**Stepwise regression models of head impact exposure.** The *x*-axis is the total number of Observed Impacts/Sessions for each player and the *y*-axis is the total number of Predicted Impacts/Sessions based on changes across all brain vital signs. *P < 0.05, **P < 0.01.

Individual pre/post-season scores are provided for each of the 15 participants (Fig. 2). Stepwise regression plots (Fig. 3) are shown for brain vital signs compared to impacts and sessions. Table 4 summarizes the stepwise linear relationships between brain vital sign and impact exposures. The highest predictive relationship was for total sessions participated (*R* = 0.86, *F* = 10.67, *P* < 0.01). Total impacts during games were also significant (*R* = 0.82, *F* = 3.78, *P* = 0.04).

## Discussion

### Main findings

The objectives of this study were to investigate the relationship between brain vital signs and HIE in youth tackle football players. The results showed significant subconcussive changes in brain vital signs, specifically in the N400 component (Fig. 1A), over the course of a season of football, replicating prior findings in ice hockey players. The N400 ERP waveform showed a ∼70ms delay in peak latency and reduced amplitude of the preceding PMN peak (Figs 1B and 2) both of which have been observed in prior studies.^16,17^ While the group-level N400 changes were most prominent, the logistic regression analysis (Fig. 3 and Table 4) demonstrated that other metrics including N100 amplitude, N100 latency and N400 latency were also significant predictors of HIE, which is also consistent with prior results.^17^

Brain vital signs data were not significantly predictive of total head impacts incurred by players over the entire season. However, when delineated by session type (practices/games), the relationship between changes in brain vital signs and impacts during games was significant. Brain vital sign changes had a more robust association with head impacts that occurred in games than practices (Fig. 3 and Table 3). This may be due to greater variability among practice impacts and/or higher impact severity in games. A novel finding was that the predictive relationship between brain vital signs and HIE extended beyond accelerometer data to include total games and practices played.

Interestingly, youth football players in this study showed more impacts in games (53.13 ± 39.91 in 9 games) than their age-group counterparts in ice hockey^17^ (32.92 ± 18.40 in 47 games) despite playing drastically less games. This impact frequency is somewhat more closely matched with the Junior-A ice hockey players (age 17–21 years). Further research should characterize these differences across sports to better understand how these factors affect subconcussive impairment.

### Functional interpretation

The N400, and the preceding PMN, have been well established in cognitive processing during speech perception.^18,19,21^ The relationships of these ERPs to existing clinical neuropsychological tests, including the Peabody Picture Vocabulary Test, Wechsler Intelligence Scale for Children III, Wechsler Adult Intelligence Scale, Psycholinguistic Assessments of Language Processing in Aphasia and the Hayling test have been thoroughly characterized in the literature.^22^ These responses have been linked to a distributed network of cortical regions involved in phonological and semantic processing in the left and right hemispheres.^19,23^ Early PMN changes have been described with respect to basic mechanisms of comprehension involved in literacy development.^24^ Indeed, lexical word access can occur as early as 200 ms.^25^ Accordingly, the specific N400 (and PMN) changes in subconcussion are of potential clinical importance.

### Potential predictive role of brain vital signs in managing subconcussion

The significant relationship between brain vital sign changes and total sessions (*R* = 0.863, *P* < 0.001) provides strong evidence that exposure to RHI throughout the season underlies the observed findings. Moreover, an association between greater HIE and larger alternations in brain vital signs suggests a predictive utility for this monitoring framework. The current results showed that N100 amplitude and N400 latency changes correlated linearly and significantly with a higher frequency of game HIEs (*R* = 0.823, *P* < 0.05). In the prior study,^17^ a similarly high predictive relationship was shown to be related to the N100 amplitude as well as the N400 latency and amplitude (*R* = 0.799, *P* < 0.01). While the current results were limited by sample size, the replicated regressions are comparably high and notably consistent. It is likely that further studies with larger samples may enable refined characterization of specific brain vital signs features that can be used prognostically. The potential for prediction has important implications for early intervention and improved treatment protocols.

### Implications of subconcussion after a single season of youth football

Previous investigations of subconcussive changes in youth football players have produced disparate results. Munce et al.^26^ found no pre/post-season deficits in clinical measures of balance, reaction time and rapid-number-naming among football players. Similarly, no adverse changes in symptoms, neuropsychological test performance, vestibular and ocular-motor screening or balance were reported for a younger group (9–12 years) of players.^27,28^ In neither study were head impact frequency or impact severity significantly related to changes in outcome measures. In a separate investigation using a battery of neuropsychological tests, head impact metrics were significantly associated with impaired performance on a list-learning task among 9- to 10-year-old football players, but no significant relationships were discovered among HIE and the other tests used.^29^ That study also found no significant associations between HIE and any measure of neuropsychological function in players aged 11–13 years.

Using MRI assessments in non-concussed youth football players (8–13 years), Bahrami et al.^8^ discovered significant relationships between HIE and decreased fractional anisotropy (FA), a measure of white matter tract activity, in multiple brain regions. In a separate investigation of white matter tract activity in youth and high school football players, there were no pre/post-season changes in FA, mean diffusivity or radial diffusivity in youth players, though they did experience reductions in axial diffusivity.^30^ Of note, high school players had significant reductions in mean diffusivity, radial diffusivity and axial diffusivity, the latter of which occurred to a greater extent than in youth players. Nilsson et al.^31^ found no pre/post-season FA changes in youth football players (8–12 years), and no differences in FA changes over time compared to age- and sex-matched swimmers. However, these investigators did find a significant relationship between one metric of HIE (magnitude of lateral head impacts) and FA in the left cingulate cortex. Negative changes in default mode network functional connectivity have recently been reported after one season in players aged 8–13 years, and these were significantly associated with players’ HIE.^32^ Collectively, pre-existing research suggests that while structural subconcussive changes in brain health can be observed using advanced neuroimaging, deficits in clinical and/or functional measures are either less common or more difficult to detect. In support of this observation, neuroimaging studies of non-concussed high school and collegiate football players have demonstrated white matter and neurophysiological changes in the absence of cognitive and neuromotor impairment.^33,34^

Findings from the current investigation are largely consistent with previous neuroimaging studies, indicating that HIE is associated with subconcussive brain changes. Presently, it is uncertain if subconcussive changes related to HIE represent (i) a benign and/or transient occurrence, (ii) an acute injury/impairment and/or (iii) predisposition to developing neurodegenerative disease (e.g. CTE). Regarding the possibility of transient abnormalities, in the study of default mode network functional connectivity by DeSimone and colleagues, they reported positive, though non-significant, changes from post-season to pre-season of the following year.^32^ These results demonstrate that brain neurophysiology is dynamic and subject to both maladaptive and adaptive changes. It is yet to be determined whether changes in brain vital signs observed in this study persist in the absence of RHI or if they resolve naturally over time.

Though acute changes in brain structure and/or function associated with RHI have not been directly linked to the pathological development of CTE, such alterations provide reasonable grounds for speculating about pathways of disease progression given that HIE has shown to be a predominant risk factor.^35^ If subconcussive changes are indeed linked to adverse health outcomes, it will be paramount to clinically evaluate and monitor football players and other contact-collision sport athletes to manage their risk. Quantified EEG approaches may provide clinicians with a portable, practical, and inexpensive method of achieving this.

### Caveats

There are a few caveats related to the interpretation of this study. First, the sample size was limited due to technical issues and player dropout such that pre–post comparisons were not possible for all participants. However, this is consistent with attrition rates in other youth football studies.^32^ Second, the clinical utility of head impact accelerometers has been questioned, given that high variability and error rates have been demonstrated between devices.^36^ The consistent relationship between impacts and sessions in this study was noteworthy, as exposure to sessions provided a comparable metric. Nonetheless, accelerometers have been shown to be useful at generalizing HIE over a longer time period.^17,36^ Third, neither concussion history nor participation in previous seasons informed the inclusion/exclusion criteria in this study. While only 20% of the study population reported a single prior concussion, this number is likely to be larger in studies with older players who have more history of contact sport participation. Future studies using larger sample sizes can evaluate these as factors. Finally, given the small sample size and homogeneous population, the generalizability of these results to other demographics such as age, sex and sport-type are unknown. Female athletes are particularly under-represented in concussion research despite demonstrating worse injury-related outcomes compared to male athletes.^37^ Ongoing work by our group and others is expanding the scope of this work accordingly.

## Conclusion

This study has demonstrated a direct link between HIE and functional brain changes in youth tackle football players using the brain vital signs framework. The findings replicated previous research in ice hockey players and added to the evidence of subconcussive brain changes associated with cumulative HIE in youth football players. The observation that more sessions of RHI were related to greater subconcussive changes within one season highlighted the importance of ongoing efforts to reduce HIE in youth football. While the clinical relevance of these findings remains to be fully characterized for both short- and long-term brain health outcomes, future research targeting larger samples of players across different age and gender groups, including longitudinal evaluations, will help explain their importance. Finally, this investigation further demonstrated the potential use of brain vital signs to monitor subconcussive brain changes in contact-/collision-sport athletes. As subconcussive brain injury becomes better characterized, the need for clinical assessments of this phenomenon is important.

## Abbreviations

CTE =: chronic traumatic encephalopathy
DTI =: diffusion tensor imaging
ERP =: event-related potential
FA =: fractional anisotropy
fMRI =: functional magnetic resonance imaging
HIE =: head impact exposure
HIT =: head impact telemetry
PMN =: phonological mismatch negativity
PoC =: point of care
RHI =: repetitive head impacts
RTP =: return to play
